# Guidance of Mesenchymal Stem Cells on Fibronectin Structured Hydrogel Films

**DOI:** 10.1371/journal.pone.0109411

**Published:** 2014-10-15

**Authors:** Annika Kasten, Tamara Naser, Kristina Brüllhoff, Jörg Fiedler, Petra Müller, Martin Möller, Joachim Rychly, Jürgen Groll, Rolf E. Brenner

**Affiliations:** 1 Laboratory of Cell Biology, Rostock University Medical Center, Rostock, Germany; 2 Division for Biochemistry of Joint and Connective Tissue Diseases of the Orthopedic Department, University of Ulm, Ulm, Germany; 3 DWI Leibniz Institute for Interactive Materials and Institute of Technical and Macromolecular Chemistry, RWTH Aachen University, Aachen, Germany; 4 Department and Chair of Functional Materials in Medicine and Dentistry, University of Würzburg, Würzburg, Germany; University of California, San Diego, United States of America

## Abstract

Designing of implant surfaces using a suitable ligand for cell adhesion to stimulate specific biological responses of stem cells will boost the application of regenerative implants. For example, materials that facilitate rapid and guided migration of stem cells would promote tissue regeneration. When seeded on fibronectin (FN) that was homogeneously immmobilized to NCO-sP(EO-*stat*-PO), which otherwise prevents protein binding and cell adhesion, human mesenchymal stem cells (MSC) revealed a faster migration, increased spreading and a more rapid organization of different cellular components for cell adhesion on fibronectin than on a glass surface. To further explore, how a structural organization of FN controls the behavior of MSC, adhesive lines of FN with varying width between 10 µm and 80 µm and spacings between 5 µm and 20 µm that did not allow cell adhesion were generated. In dependance on both line width and gaps, cells formed adjacent cell contacts, were individually organized in lines, or bridged the lines. With decreasing sizes of FN lines, speed and directionality of cell migration increased, which correlated with organization of the actin cytoskeleton, size and shape of the nuclei as well as of focal adhesions. Together, defined FN lines and gaps enabled a fine tuning of the structural organization of cellular components and migration. Microstructured adhesive substrates can mimic the extracellular matrix *in vivo* and stimulate cellular mechanisms which play a role in tissue regeneration.

## Introduction

Fate and function of stem cells are controlled by adhesive interactions with the extracellular matrix mediated by adhesion receptors, like integrins [1]. Therefore, the design of a material that serves as a substrate for cells and specifically determines survival, proliferation, differentiation, and migration is a great challenge for application in regenerative medicine. To regenerate bone, cartilage or other tissues of the mesenchyme after injury or disease a suitable scaffold incorporated at the site of injury *in vivo* could provide components of a stem cell niche that promote the activity of mesenchymal stem cells (MSC) which can be transplanted together with a scaffold or recruited from bone marrow. Beside of the type of extracellular matrix or components of matrix proteins, designing defined topographies as adhesive substrate to control cell shape has demonstrated the commitment of MSC to develop to an adipocyte or osteoblast [2]. In addition, nanofeatures of surface topographies, differing in ordered or disordered patterns controlled the differentiation of MSC to osteoblasts or facilitated self-renewal [3]–[5]. Beside multiple differentiation and self-renewal of adult stem cells, directed migration of stem cells is fundamental for tissue formation and regeneration [6]. Although a number of investigations have revealed detailed mechanisms of cell migration, little is known how the migration of MSC can be controlled by tailored material surfaces which can be used as implants. How a surface with defined structures for cell adhesion controls migration of stem cells is poorly understood [7]. The controlled guidance of stem cell migration by a material surface would have significant implications for regenerative medicine. Stimulation of migration can disperse the stem cells, which have been transplanted into the body to the surrounding tissue for regeneration. Materials could also be used to stimulate the recruitment of stem cells, which already exist in the body to the desired anatomic site.

In order to allow cells only to adhere via FN interactions, we first covered the surface with a thin layer of the star shaped polymer NCO-sP(EO-*stat*-PO) hydrogel which acts as a protein repellent and primarily inhibits cell adhesion as described earlier [8]–[10]. FN was then immobilized onto this layer. First we tested whether FN homogeneously immobilized using this technique modulates adhesion mediating components and migration of MSC compared to a glass surface as control. Using soft lithography we then created defined lines of FN with different sizes. The spacings between the lines prevented adhesion of cells. Because cell migration is controlled by mechanisms involved in cell adhesion, like formation of focal adhesions or organization of the actin cytoskeleton we tested whether these parameters correlated with the patterns of FN lines and with cell migration. In addition, we evaluated the morphology of cells and how the shape of the nucleus was controlled by the lines. Our results demonstrate that the width of FN lines allowing cell adhesion and spacings are suitable to control different cellular components involved in cell adhesion and enables a fine tuning of speed and orientation of cell migration.

## Materials and Methods

### Ethics statement

The study was approved by the Ethics Committees of the Rostock University Medical Center (A21/207) and the University of Ulm (242/2004). A written consent for using the samples for research purposes was obtained from all patients prior to surgery.

### Preparation of surfaces

Glass cover slips were coated with NCO-sP(EO-*stat*-PO) as reported earlier [10], [11]. Briefly, substrates were cleaned by exposure to UV/ozone for 10 min and subsequently aminofunctionalized through silanization. NCO-sP(EO-*stat*-PO) was dissolved in dry tetrahydrofuran and diluted with water 5 min prior to spin-casting to a solution with a concentration of 10 mg/ml and 90 Vol% water. As negative controls to demonstrate that no cells adhere on plain NCO-sP(EO-*stat*-PO) coated substrates, such films were stored for 24 hours under ambient conditions and then used further. For non-patterned functionalization of the coatings with FN, freshly NCO-sP(EO-*stat*-PO) coated glass substrates were allowed to dry under the clean bench for 1 h under ambient conditions and subsequently incubated with FN from human plasma for 20 min using 50 µg/ml FN solution in water. This leads to covalent attachment of the protein to the substrate, because freshly prepared NCO-sP(EO-*stat*-PO) layers remain NCO-functional, as hydrolysis and subsequent aminolysis of the isocyanates take several hours [12]. After washing with water, the films were then stored under ambient conditions over night prior to further use. Non-coated glass cover slips served as non-coated control substrates.

For pattern formation, polydimethylsiloxane (PDMS) microstructured stamps were generated through curing of Sylgard 184 on negatively structured silicon masters. The stamps were incubated with FN solution (50 µg/ml in water) for 15 minutes each, dried under nitrogen stream and subsequently set onto the substrates without any pressure and remained there for 20 min. After removal of the stamps, the substrates were stored under ambient conditions over night prior to further use. Lines in various sizes (10–80 µm) and spacing dimensions (5–20 µm) were fabricated. Unless otherwise stated, the following line and spacing dimensions (fibronectin line width [µm]/spacing size [µm]) were used: FN 10/10, FN 20/10, FN 50/20 and FN 80/20. To visualize the fibronectin lines, a primary antibody against FN (Sigma-Aldrich Chemie GmbH, Steinheim, Germany) as well as Alexa Fluor 488-conjugated anti-rabbit IgG (Life Technologies GmbH, Darmstadt, Germany) as secondary antibody were used.

### Cell culture

Human mesenchymal stem cells (MSC) were isolated from bone marrow obtained during either median sternotomy or pelvic osteotomy. Together, bone marrow from 20 donors, aged 15 to 80 years, both female and male were included in the experiments (see table S1). According to a standard protocol as described earlier [13], cells were enriched using density gradient centrifugation of the diluted marrow sample (d−1.077 g/l) and cultured at 37°C and under 5% CO_2_ for 24 h in Dulbecco's modified Eagle's medium supplemented with 1% antibiotic-antimycotic solution (both from Life Technologies GmbH) and charge tested 10% fetal calf serum (FCS) (PAN-Biotech GmbH, Aidenbach, Germany). Adherent cells were harvested and the purity of MSC was proven by their ability to differentiate into both osteoblasts and adipocytes. Cells were grown in cell culture flasks until passage 3 before introducing them into the experiments. For the experiments, cells were detached with 0.05% trypsin/0.02% EDTA (Sigma-Aldrich Chemie GmbH) and seeded with a density of 3×10^3^ cells/cm^2^ onto homogeneously functionalized cover slips or with a density of 6×10^3^ cells/cm^2^ onto patterned cover slips unless otherwise stated.

### Staining of cytoskeletal structures, nuclei and cell membranes

Cells on cover slips were fixed with 4% paraformaldehyde and permeabilized with 0.1% triton X-100 (both from Sigma-Aldrich Chemie GmbH) for 10 min. To visualize focal adhesions, cells were incubated with a monoclonal antibody against vinculin (clone hVIN-1; Sigma-Aldrich Chemie GmbH) or a polyclonal antibody against paxillin (clone H-114; Santa Cruz Biotechnology, Inc., Santa Cruz, CA) for 4 h at room temperature (RT) followed by incubation with a secondary antibody against mouse or rabbit IgG labeled with Alexa Fluor 488 (both from Life Technologies GmbH) for 30 min in the dark at RT, respectively. All antibodies were used at a dilution of 1∶100 in phosphate-buffered saline (PBS; PAA laboratories, Pasching, Austria). To visualize the F-actin cytoskeleton, Alexa Fluor 546 phalloidin (Life Technologies GmbH) was used.

To analyze spreading of cells, cell membranes were stained using the PKH26 Red Fluorescent General Cell Linker Kit (Sigma-Aldrich Chemie GmbH) according to the manufacturer's instructions. This dye enables staining of the complete cell membrane to visualize cells in a fluorescence mode. Briefly, detached cells were suspended in a staining solution and then incubated for 5 min at 37°C. The staining reaction was stopped by addition of FCS. Cells were centrifuged, washed twice with PBS, resuspended in culture medium, and seeded onto homogeneously functionalized cover slips providing only a defined area of adhesion by using flexiPERM con A rings (Sarstedt, Nümbrecht, Germany) with a diameter of 12 mm. For staining of nuclei, spread cells were incubated with 1 µg/ml Hoechst 33342 (OmniChem, Louvain-la-Neuve, Belgium) diluted in cell culture medium for 15 min at 37°C.

After staining procedures, coverslips were embedded in mounting medium consisting of 30% glycerine (w/v; Merck, Darmstadt, Germany), 12% polyvinylethanol (w/v; Sigma-Aldrich Chemie GmbH), 0.53 mM phenol, and 60 mM TRIS buffer (both from Carl Roth, Karlsruhe, Germany) in distilled water.

### Microscopical examinations

The morphology of MSC cultured on the fibronectin lines was evaluated 24 h after seeding using an Axiovert microscope (Carl Zeiss, Jena, Germany). To microscopically analyze the organization of the actin cytoskeleton, the formation of focal adhesions as well as for visualizing the fibronectin stripes, a Leica TSC SP2 AOBS confocal laser scanning microscope (Leica Microsystems GmbH, Wetzlar, Germany) was used. An Observer.Z1 fluorescence microscope (Carl Zeiss) was used to visualize cell membranes and nuclei of cells.

### Determination of morphometric parameters

The image processing software ImageJ version 1.44p (NIH software, USA) was used to determine the orientation of nuclei and cells as well as the projected area and perimeter of cells, nuclei, and focal adhesions. The form factor was calculated as 4π*area/perimeter^2^. A form factor of 1.0 describes a perfectly round cell, whereas a form factor lower than 1.0 represents a polygonal and more elongated cell [14].

### Cell migration

To evaluate cell migration, 5×10^3^ cells were seeded onto cover slips prepared as described above, i. e. homogeneously functionalized surfaces or micro-patterned surfaces (line width [µm]/spacing sizes [µm] of 10/5, 20/10, 50/20 and 80/20) and placed in a well of a 12-well plate. After allowing cells to adhere for 10–18 h, images of cells were recorded every 30 min on an Olympus IX81 motorized inverted microscope (Olympus, Hamburg, Germany) equipped with the GP168 II incubator from EMBL Heidelberg with CO_2_ delivery. Images were analyzed using Cell(R) software software (Olympus Europe, Hamburg, Germany). After 24 h all pictures were automatically assembled to a continuous movie using the Cell(R) software. Overall, the tracks of 10 cells per movie were analyzed to determine both velocity and orientation of migration using the ImageJ software with an additional Chemotaxis and Migration Tool (ibidi, Martinsried, Germany).

### Statistics

Statistical analyses were performed using SPSS 15.0 software (SPSS, Inc., Chicago, IL). Kolmogorov-Smirnov test was used to test normal data distribution. Because data were not normally distributed, the Mann-Whitney U-test was used. Significant differences were established at three levels (* *P*<0.05, ** *P*<0.01, *** *P*<0.001). For analyses of areas and form factors, cells of one donor were used. For analyses of migration, cells of 7 (homogeneous surfaces) or 8 (on lines) donors were used. In each case the number of cells (n) is indicated. For the evaluation of focal adhesions, 40 adhesions per cell were analyzed. All graphs were created using SigmaPlot 11.0 software (Systat Software, Inc., San Jose, CA). Graphs display box-and-whisker diagrams unless otherwise stated. Boxes include 25^th^ and 75^th^ percentiles as well as the median. Whiskers represent 10^th^ and 90^th^ percentiles. All values mentioned in the text are presented either as median values or in case of the orientation angle of cells as mean values ± standard deviation.

## Results

### Homogeneously functionalized substrates

#### Cell spreading, and organization of components of cell adhesion and cytoskeletal structures

**Figure 1:**
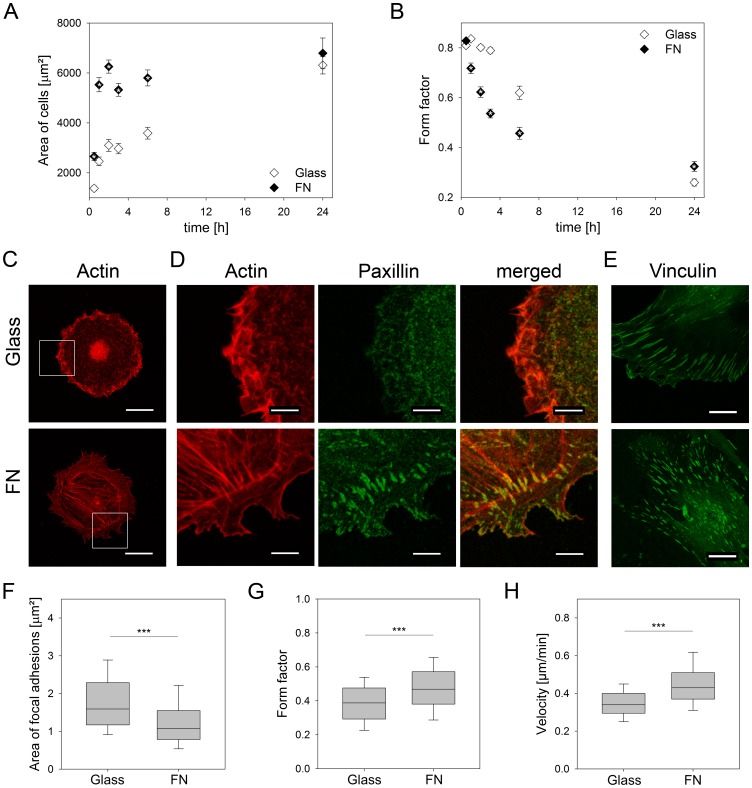
Characterization of cell parameters on homogeneous fibronectin coatings. (A) Cell area and (B) form factor were determined at several time points within 24 h after cells were plated on fibronectin or glass. Up to 6 h after plating, cells spread faster and gained earlier their final shape on fibronectin than on glass. After 24 h similar values were obtained on both surfaces. (C, D) Cells were stained for F-actin and paxillin 1 h after cell seeding to detect the actin cytoskeleton and focal adhesions, respectively. White squares in (C) outline the regions shown enlarged in (D). On fibronectin, F-actin organized into circular bundles as well as dorsal stress fibers which ended in focal adhesions. To characterize the focal adhesions on fibronectin (E) cells were stained for vinculin. (F) Area and (G) form factor of focal adhesions were analyzed. The largest and most elongated focal adhesions were determined on glass compared to cells on fibronectin. (H) On fibronectin, cells migrated faster than cells on glass. (A, B) n = 45, mean ± SEM, (F, G) n = 6, (H) n = 170. *P*-values are denoted by asteriks: *P<0.05, **P<0.01, ***P<0.001. Scale bars: (C) 23.81 µm, (D) 6.8 µm, (E) 15.87 µm.

First we have been interested whether homogeneously immobilized fibronectin differentially regulate mechanisms of cell adhesion and the organization of cytoskeletal structures compared to a glass surface as control. On FN cells did adhere and spread significantly faster than on glass (Fig. 1A). By analyzing the form factor, we found that FN strongly supported the formation of a final elongated and polygonal shape (Fig. 1B). Similarly, the formation of the actin cytoskeleton and focal adhesions (Fig. 1C–D) was significantly more stimulated on FN than on the control substrate. Within one hour, cells on FN were able to organize a structured actin cytoskeleton consisting of arched and circular bundles as well as dorsal stress fibers. In contrast to cells on glass, paxillin as a component of focal adhesions co-localized with actin on FN confirming that dorsal stress fibers end in focal adhesions. Next we analyzed size and shape of focal adhesions after 24 h (Fig. 1E–G). Focal adhesions on FN (1.074 µm^2^), were smaller than detected on glass (1.592 µm^2^). Shape analysis of focal adhesions revealed a rounder shape (form factor 0.47) on FN compared with cells on glass (0.39).

#### Cell migration

Because migration of stem cells is a significant function in regenerative processes, we tested whether the differences we found regarding the formation of cytoskeletal structures and other components of cell adhesion in dependance on the substrates correlate with cell migration. The results revealed that cells on FN migrated faster (0.43 µm/min) than on glass (0.34 µm/min) (Fig. 1H).

### Micro-patterned lines

**Figure 2:**
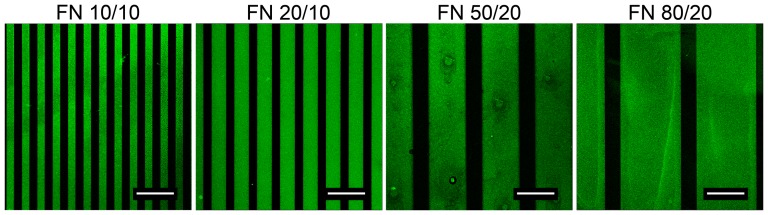
Visualization of linear micro-patterned fibronectin. Cell repellent NCO-sP(EO-*stat*-PO) coatings were linearly micro-patterned with fibronectin. Line dimensions ranged from 10 µm to 80 µm. Fibronectin lines were stained with an anti-fibronectin antibody (green). (Scale bars: 47.62 µm) (FN 0/0 = Fibronectin, line width/spacing size).

Based on the results demonstrating a superior effect of fibronectin compared to an uncoated glass surface on cell adhesion and migration, micro-patterned lines of fibronectin on primary cell repulsive NCO-sP(EO-*stat*-PO) covered surfaces were used to investigate cellular responses (Fig. 2).

#### Cell orientation

**Figure 3:**
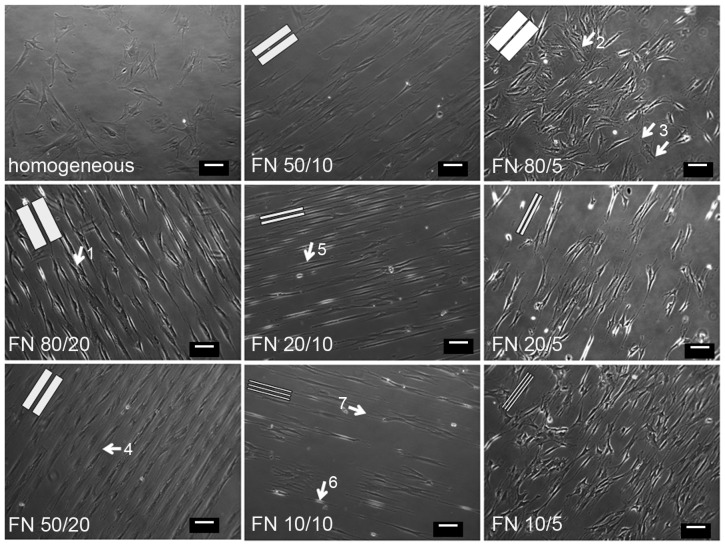
Cell morphology on various line and spacing dimensions. Widths of fibronectin lines and spacings are shown in the images (FN line width/spacing size). Cell morphology was analyzed 24 h after cells were plated on homogeneous fibronectin or micro-patterned substrates. Cell morphology was clearly dependent on both line width and spacings. Main observations are indicated by white arrows. Two cells are able to adhere adjacent to each other on both 80 µm (1) and 50 µm (4) lines. Due to a reduction of line spacings up to 5 µm, cells are able to bridge non-adhesive spacings and may thereby loose alignment along the line direction (2). Furthermore, the cells formed more protrusions in different directions than on homogeneously functionalized fibronectin (3). In dependancy on the line width, cells were able to bridge the critical distance of 10 µm between lines. On 20 µm lines, the cells were elongated and rarely bridged non-adhesive 10 µm spaces (5). On 10 µm lines, cells were mostly elongated (6), but compared to 20 µm wide lines, cells were more often able to bridge several 10 µm non-adhesive spaces and had multiple protrusions (7). White Bars represent dimensions of fibronectin lines to scale, respectively.

**Figure 4:**
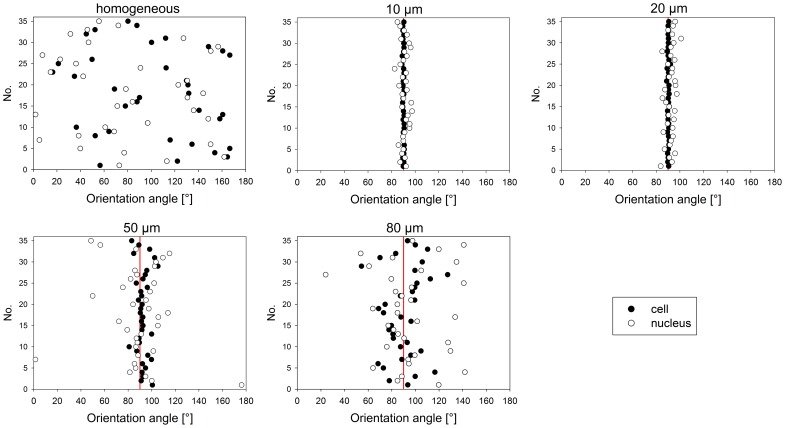
Alignment of cells and nuclei on various line dimensions. Orientation angle (ranging from 0° to 180°) was measured using a horizontal line as reference line. On micro-patterned substrates, orientation angle of stripes was 90° (indicated by red lines). On 10 µm and 20 µm lines, the orientation of cells and nuclei followed strictly the orientation of micro-patterned lines. These effects were less pronounced on 50 µm and 80 µm lines. On homogeneously functionalized fibronectin substrates, both cells and nuclei were randomly oriented with no preferred direction.

The behavior and morphology of cells were analyzed (Fig. 3), i.e. whether cells (1) are organized as single cells behind each other or have contact to neighboring cells by staying abreast, (2) can bridge the gaps between fibronectin lines, and (3) align along the lines and to what extent.These parameters were found to be dependent on both line and spacing dimensions. 80 µm lines and 50 µm lines allowed adhesion of two or three cells in parallel enabling the formation of cell-cell contacts. On smaller lines, single cells formed a line and had no contact to neighboring cells. Furthermore, line and spacing dimensions determined whether cells were able to bridge the non-adhesive spacings. Spacings of 5 µm and 10 µm were frequently spanned by cells, which was not obvious on broader lines of 20 µm. In general, cross-bridging of a gap was accompanied by an increased number of protrusions as well as a reduction or loss of cell alignment along the line direction. The orientation of cells in terms of random distribution or alignment along the line direction was highly controlled by line width. Quantitative analysis of cell alignment (Fig. 4) revealed randomly oriented cells with no preferred direction on homogeneously functionalized FN substrates (100.8°±46.3°), whereas FN lines forced the cells to align along the line direction (90°) resulting in a slight alignment on 80 µm lines (90.3°±15.6°) and a drastic alignment on 10 µm lines (90.0°±0.5°) and 20 µm lines (90.2°±0.6°). These findings indicate an inverse correlation between line width and the degree of alignment. In general, orientation of nuclei corresponded with the orientation of cell bodies, although to a lesser extent. On homogeneously functionalized FN, nuclei were randomly distributed (81.0°±49.2°).

#### Size and shape of cells and nuclei

**Figure 5:**
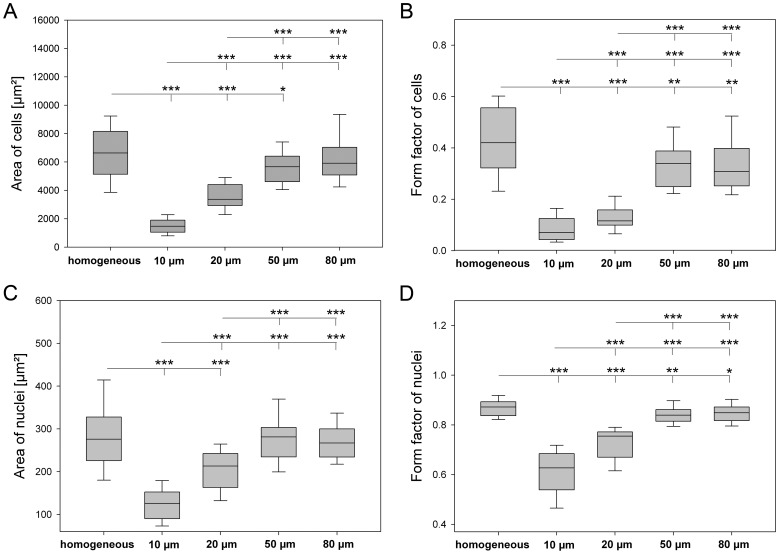
Regulation of morphometric parameters of cells (A, B) as well as nuclei (C, D) on various line dimensions. 24 h after cell seeding, morphometric parameters such as area (A, C) and form factor (B, D) were determined. When cells were seeded on micro-patterned lines, a drastic elongation of both nuclei and cells was observed with the highest extent on 10 µm lines. Similarly, the size of cells and nuclei was dependent on line width. Size of cell and nuclear areas correlated with decreasing line width. (n = 35, *P*-values are denoted by asterisks: * *P*<0.05, ** *P*<0.01, *** *P*<0.001).

In dependancy of line width, size and shape of cells (Fig. 5A–B) as well as nuclei (Fig. 5C–D) were quantified. Cells reached a maximal size on 80 µm lines (6,116 µm^2^) as well as on homogeneously functionalized FN substrates (6,635 µm^2^). A significant reduction of cell size correlated with a decrease in line width resulting in a minimal cell size on 10 µm lines (1,665 µm^2^). Moreover, cells became more elongated with decreasing line width. On 10 µm lines, cells had a maximal spindle-like shape displayed by a form factor of 0.071 in contrast to significantly increased form factors of cells on 80 µm lines (0.309) and homogeneous fibronectin (0.42). Similarly, nuclear size and shape was influenced by line width resulting in the smallest and most round shaped nuclei on 10 µm lines with an area of 126 µm^2^ and form factor of 0.63 compared to 80 µm lines (area: 270 µm^2^, form factor: 0.84) and homogeneous FN (area: 276 µm^2^, form factor: 0.87).

#### Cytoskeletal structures

**Figure 6:**
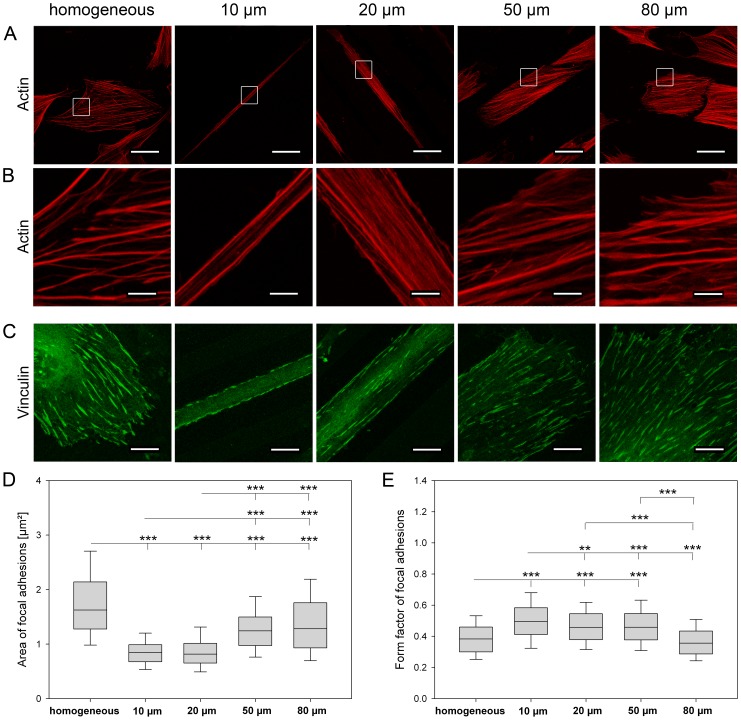
Organization of the actin cytoskeleton and chracterization of focal adhesions on various line dimensions. (A) Staining for F-actin (red) in control cells on homogeneous fibronectin and in cells on fibronectin lines ranging from 10 µm to 80 µm. White squares outline the regions shown enlarged in (B). Notably on 10 µm and 20 µm lines, actin stress fibers were oriented along the direction of the micro-patterned line. With increasing line width, the degree of alignment was decreased. In general, number of actin stress fibers was reduced due to decreasing line width. (C) Focal adhesions were visualized using an anti-vinculin antibody. (D) Area and (E) form factor of focal adhesions are shown. On 10 µm lines, focal adhesions were linearly oriented on line borders. Focal adhesions became smaller and more elongated with decreasing line width. (Scale bars: (A) 47.62 µm, (B) 6.8 µm, (C) 11.9 µm; (D, E) n = 6, *P*-values are denoted by asterisks: * *P*<0.05, ** *P*<0.01, *** *P*<0.001).

Because cell shape has shown to be dependent on line width, we further asked whether the actin cytoskeleton as well as focal adhesions were differently organized in dependance on line sizes and spacings (Fig. 6). We found that a decreasing line width was accompanied by a reduced number and a more directed orientation of the actin filaments along the line direction. Obviously, the degree of reorganization of the actin cytoskeleton increased with decreasing line width. To visualize and quantify size and shape of focal adhesions, vinculin as a component of focal adhesion was stained. Cells on homogeneously functionalized FN substrates developed the largest focal adhesions (1.626 µm^2^) whereas the size of focal adhesions decreased gradually from 80 µm lines (1.283 µm^2^) to 10 µm lines (0.846 µm^2^). The shape of focal adhesions revealed a most elongated shape on 80 µm lines (form factor 0.356) and homogeneous FN (0.382), and the roundest shape on 10 µm lines (0.495). Similar to the highest degree of reorganization of the actin cytoskeleton on 10 µm lines with only two prominent orientated actin fibers, focal adhesions on 10 µm lines were likewise distributed along line borders.

#### Cell migration

**Figure 7:**
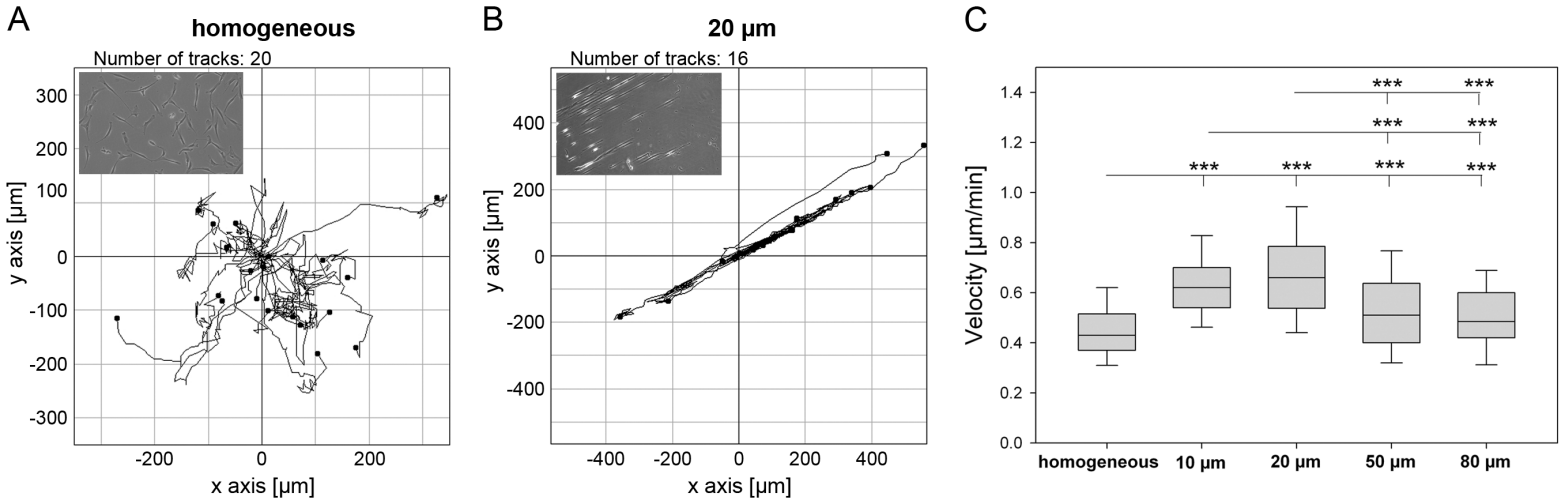
Regulation of cell migration by linear surface patterning. The following line and spacing dimensions (fibronectin line width [µm]/spacing sizes [µm]) were used: FN 10/5, FN 20/10, FN 50/20 and FN 80/20. For time-lapse microscopy of migrating cells, cells were tracked on (A) homogeneous fibronectin and (B) 20 µm fibronectin lines with a spacing of 10 µm. Cells on 20 µm lines migrated strictly along the line direction, whereas cells on homogeneously functionalized fibronectin substrates migrated randomly. (C) The highest migration rate was determined in cells on 10 µm and 20 µm lines. (n = 90, *P*-values are denoted by asterisks: * *P*<0.05, ** *P*<0.01, *** *P*<0.001).

Because parameters of cell adhesion were affected by micro-patterned substrates, we tested whether these findings correlated with both velocity and directionality of cell migration (Fig. 7). In general, cells migrated faster on FN lines than on homogeneous FN surfaces (0.43 µm/min). Moreover, cell migration was dependent on line width. The migration speed increased with decreasing line size (0.5 µm/min, 0.62 µm/min, 0.66 µm/min for lines sizes of 80 µm, 20 µm, 10 µm, respectively). The directionality of migration was also determined by the substrate patterns. As shown in Fig. 7, cells on homogeneous FN migrated randomly in all directions. In contrast, cells on 20 µm lines migrated highly directional. However, the reduction of spacing dimensions to 5 µm resulted in a strongly reduced directionality due to the bridging of cells over several lines (not shown).

## Discussion

As shown earlier, surface coating with the star shaped polymer NCO-sP(EO-*stat*-PO) preserves the biological functionality of integrated proteins or peptides, as e. g. mediates cell adhesion, even on deformable substrates under mechanical deformation. [15], [16]. Here we focused on cell migration as a crucial function of mesenchymal stem cells in regenerative processes. To test, whether immobilization of FN on the star shaped polymer is functionally active, our results provided evidence that MSC on FN induced a higher rate of migration than on a glass surface. Migration of cells is facilitated by the dynamic adhesive interaction of cells with the substrate, which is determined by specific integrin-ligand binding affinity, level of ligands, generation of forces at binding sites [17]. The coating with NCO-sP(EO-stat-PO) we used to immobilize FN generated layer thicknesses below 40 nm. At this thickness an influence of a mechanical difference by the coating compared with glass as control can be excluded and we suggest that the mechanical characteristics are determined by the substrate beneath, which is glass in both cases. Other experiments have demonstrated that beside the chemical structure of the ligand, the mechanical property of the matrix determines cell migration [18], [19]. On FN we revealed a faster cell spreading, an enhanced formation of the actin cytoskeleton and its co-localization with proteins found in focal adhesions. These dynamic processes are mediated by molecular activities in focal adhesions. Our results have shown that on FN focal adhesions were smaller and revealed a more round shape compared with cells on glass. The size of focal adhesions can be controlled by the chemistry of the substrate or its mechanical characteristics, because focal adhesions are force-sensitive structures and the turnover of the proteins inside are controlled by mechanical forces [20]. In subsequent experiments to exlore how differently sized lines of FN and also varying non-adhesive spacings control the behavior of cells, we first found that the morphology and the organization of cells varied with the size of FN lines. On larger 80 µm lines, cells formed cell-cell contacts, which enables a collective migration. Collective cell migration, rather than individual migration, is characteristic for physiological processes in the organism, like morphogenesis and tissue repair [21]. The migration in cell sheets is determined by a mechanical coupling between the cells, facilitated by specific cell-cell junctions that depend on the cell type [22]–[24]. In context with tissue engineering approaches, our surfaces providing broad lines for cell adhesion, would be suitable to transplant and guide for example a co-culture of cells for tissue regeneration. In a previous study we have demonstrated that osteogenic differentiation of mesenchymal stem cells was stimulated in a co-culture by direct cell contacts to endothelial cells [25]. With decreasing sizes of the lines in our experiments, individual cells without contact to neighboring cells covered the FN lines and with smaller distances between the lines cells bridged the lines above non-adhesive gaps. In cells forming bridges between adhesive areas, increased traction forces at the edge of the adhesive sites have been demonstrated [26]. These alterations of intracellular forces are facilitated by strong actin filaments crossing the bridges and a dynamic movement of the actomyosin molecules [26]–[28]. Force generation by the cells is required to organize the extracellular matrix [29]. By fabrication of substrates with line patterns that allow cell bridging over non-adhesive gaps we mimic the situation *in vivo*. The architecture of the extracellular matrix *in vivo* is comprised of a network of fibers to which cells adhere. To cross the micron-sized gaps inside the filamentous network, cells have to form bridges.

By further evaluating the organization of intracellular components of cell adhesion our data clearly indicate that the geometry of the environment was translated into the organization of subcellular structures. The actin filaments became strongly aligned with decreasing FN lines, focal adhesions decreased in size and became more round shaped. The formation and size of focal adhesions is connected with the actin cytoskeleton that controls the size of focal adhesions by forces mediated by myosin IIA [30]. In addition to the size and shape of focal adhesions, the function of defined proteins in focal adhesions and the turnover of proteins, i.e. shuttling between adhesions and the cytosol are controlled by the geometric constraints of the cellular environment and correlate with functional activities. For example, on islands of extracellular matrix, paxillin has been shown to localize the activated signaling protein Rac to form lamellipodia [31]. On small FN lines compared with a 2D matrix, vinculin and paxillin demonstrated a decreased turnover within focal adhesions, which indicates a prolonged adhesive contact with the substrate [32].

Our results revealed a differential control of the shape, both of the total cell and the nucleus by the width of the FN lines. While convincing evidence exists that the shape of MSC commits the direction of differentiation and controls cellular self-renewal [33], studies on the impact of deformation of the nucleus by physical cues are rare. *In vivo*, deformation of the nucleus is controlled by the stiffness of the tissue or when cells migrate through a dense extracellular matrix [34]. Changes in the nuclear shape induced by the stiffness of the matrix or by microfabricated surfaces induced an osteogenic differentiation [35], [36]. In accordance with our results, fibroblasts on microgrooved surfaces generate an elongated shape of the nucleus [37]. The impact of a biophysically induced shape change of the nucleus concerning a biological cell response was highlighted by the finding that the efficiency of the reprogramming of fibroblasts to pluripotent stem cells was improved [37]. Deformation of the nucleus is connected with alterations in the structure of chromatin which is involved in mechanisms of epigenetic modifications in somatic cells, such as histone H3 acetylation and methylation [37].

To correlate the structural alterations of subcelluar components with functional activities of MSC we have focussed on cell migration. In contrast to a homogeous FN surface we observed a directed migration on FN lines and with decreasing line width the migration rate increased. The lower migration speed on 10 µm compared with cells on 20 µm lines suggests that the observed crossing of the lines inhibits the migration speed. Concerning the mechanisms how the speed of migration is regulated there is a general consensus that myosin-II-dependent traction forces determine the mechanical interaction with the substrate and sense the geometric dimensions [38]. Lower traction forces were found when fibroblasts were cultured on very small 1D lines compared with cells on a homogeneous 2D substrate, which would enable a faster migration on a 1D line [39]. Another study revealed a prolonged and increased mechanical coupling between the cytoskeleton and the focal adhesions, which allows a greater protrusion at the leading edge of the cell to move forward [32]. Our experiments revealed that cells not only distinguish between 2D and 1D, but MSC are sufficiently sensitive to sense various sizes of lines and spacings to control cell migration. Thus, designing appropriate line sizes and spacings enables the tuning of migration speed and a directed collective migration to form multicellular structures to regenerate tissues of the mesenchyme.

## Conclusions

The results highlight the impact of the generation of material surfaces with specifically interacting adhesive structures for applications to regenerate tissues. By tuning line structures of FN on otherwise cell repellent surfaces we were able to control the organization of subcellular components and migration of cells. At least to some extent we are able to mimic adhesive interactions of MSC *in vivo*, where the extracellular matrix enables collective cell migration or the collagen network forces cells to bridge the collagen fibers. We provide evidence that MSC sense different line sizes and gaps which enables a subtle governing of cellular components to control physiological processes like cell migration.

## Supporting Information

Table S1 **Donor information related to individual experiments.**
(DOCX)
