# Supplementary material for: Guidance of Mesenchymal Stem Cells on Fibronectin Structured Hydrogel Films
Source: PLoS One. 2014 Oct 15;9(10):e109411. doi: 10.1371/journal.pone.0109411 (PMC4198140; doi:10.1371/journal.pone.0109411)
Supplement: Table S1 — Donor information related to individual experiments. (DOCX) [file pone.0109411.s001.docx]

**Supporting Information**

Table S1. **Donor information related to individual experiments**

| **Experiment** | **No. of donors** | **Gender** | **Age (years)** | **Disease** |
| --- | --- | --- | --- | --- |
| Fig. 1A, 1B, 1C | 1 | male | 66 | Triple vessel disease |
| Fig. 1F, 1G | 1 | male | 80 | Triple vessel disease |
| Fig. 1H | 7 | 2 male, 5 female | 15-44 (mean 32) | Triple osteotomy |
| Fig. 3 | 1 | male | 69 | Triple vessel disease |
| Fig. 4, Fig. 5A-D | 1 | male | 55 | Triple vessel disease |
| Fig. 6D-E | 1 | male | 52 | Triple vessel disease |
| Fig. 7C | 8 | 1 male, 7 female | 23-42 (mean 29) | Triple osteotomy |
